# Supplementary figures and images for: Differential Expression of miRNAs, lncRNAs, and circRNAs between Ovaries and Testes in Common Carp (Cyprinus carpio)
Source: Cells. 2023 Nov 15;12(22):2631. doi: 10.3390/cells12222631 (PMC10670750; doi:10.3390/cells12222631)

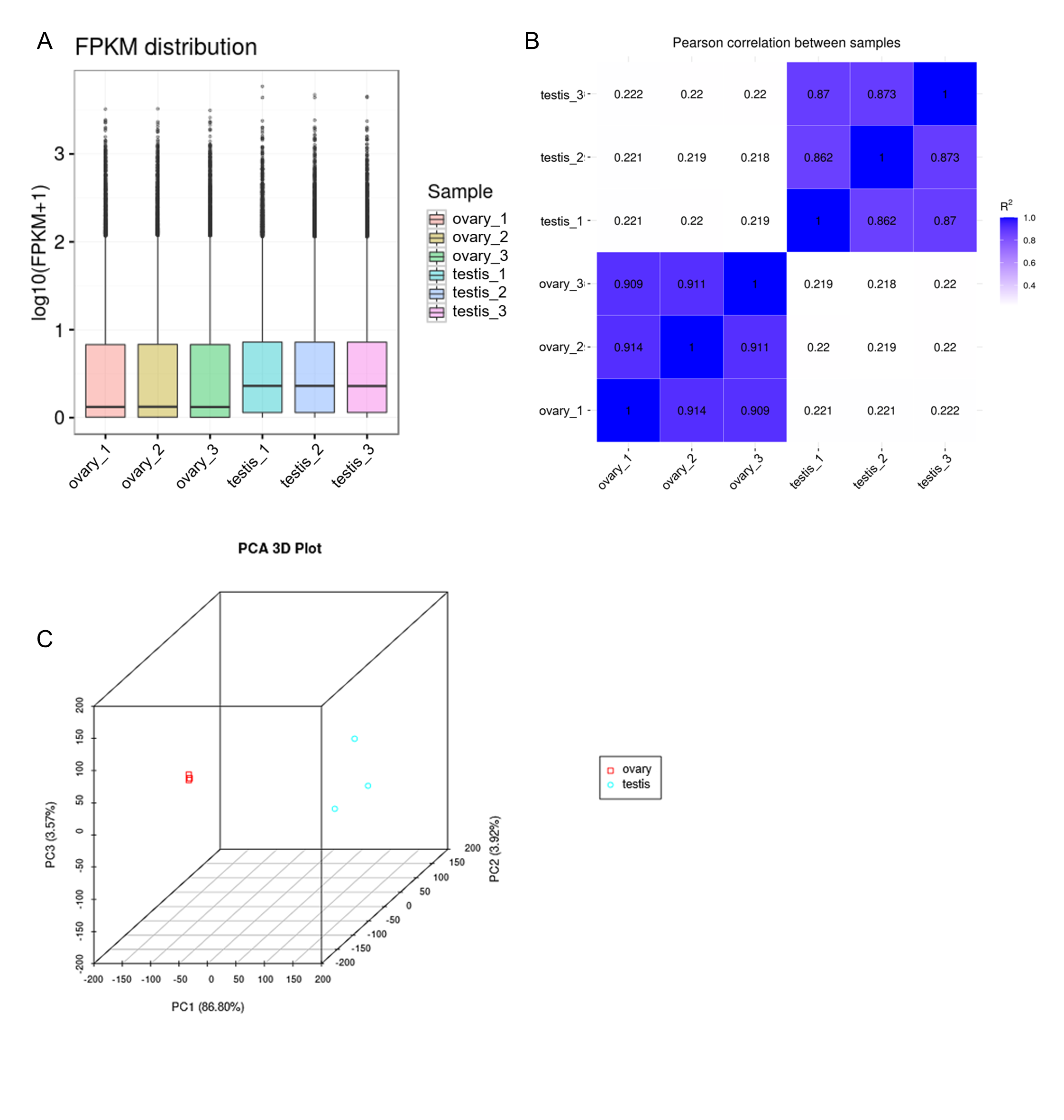

Supplement: Supplementary file 1 [file cells-12-02631-s001.zip › Figure S1. Expression and correlation analysis of each sample.tif]

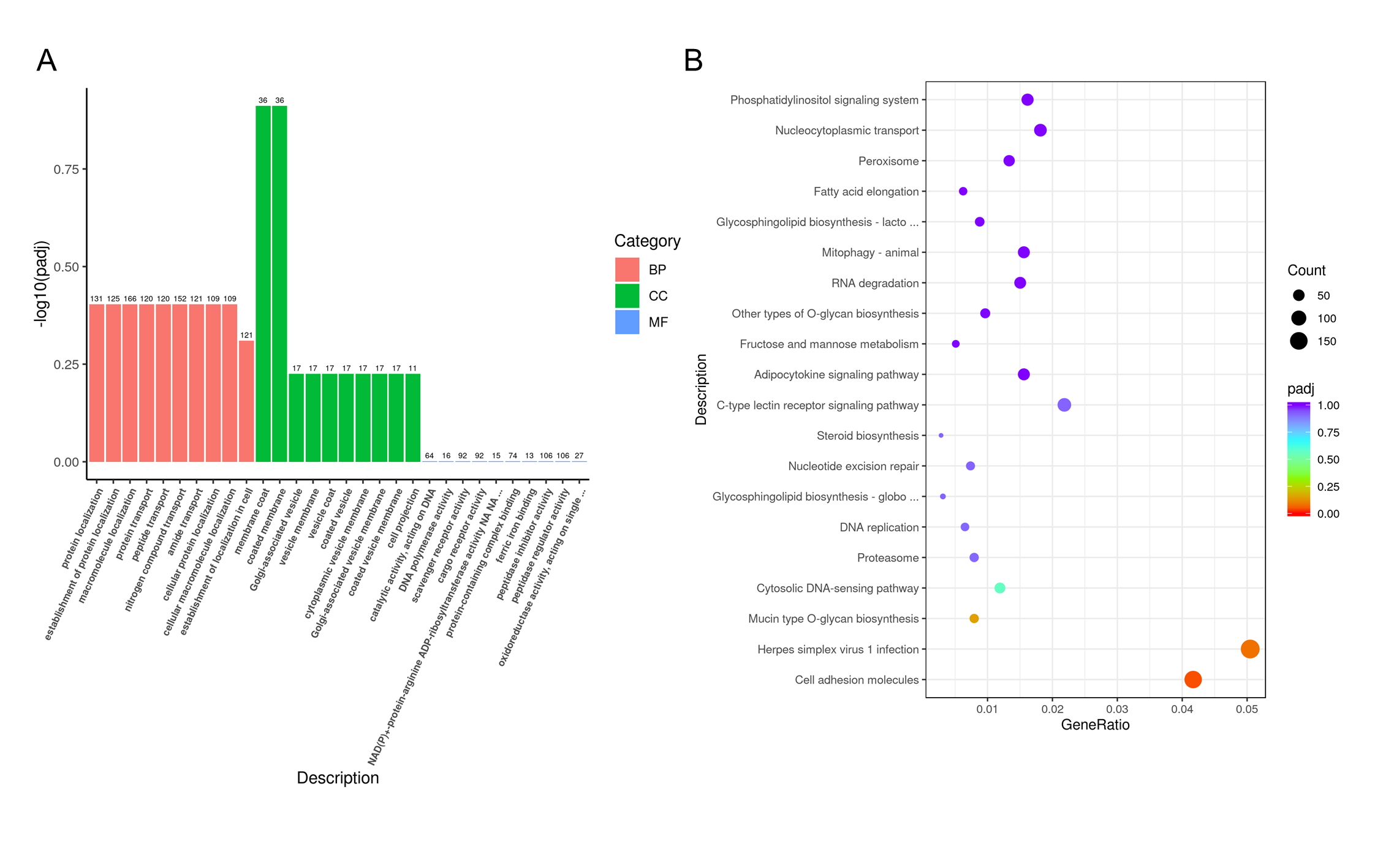

Supplement: Supplementary file 1 [file cells-12-02631-s001.zip › Figure S2. Enrichment of lncRNA target genes predicted from co-location analysis.tif]

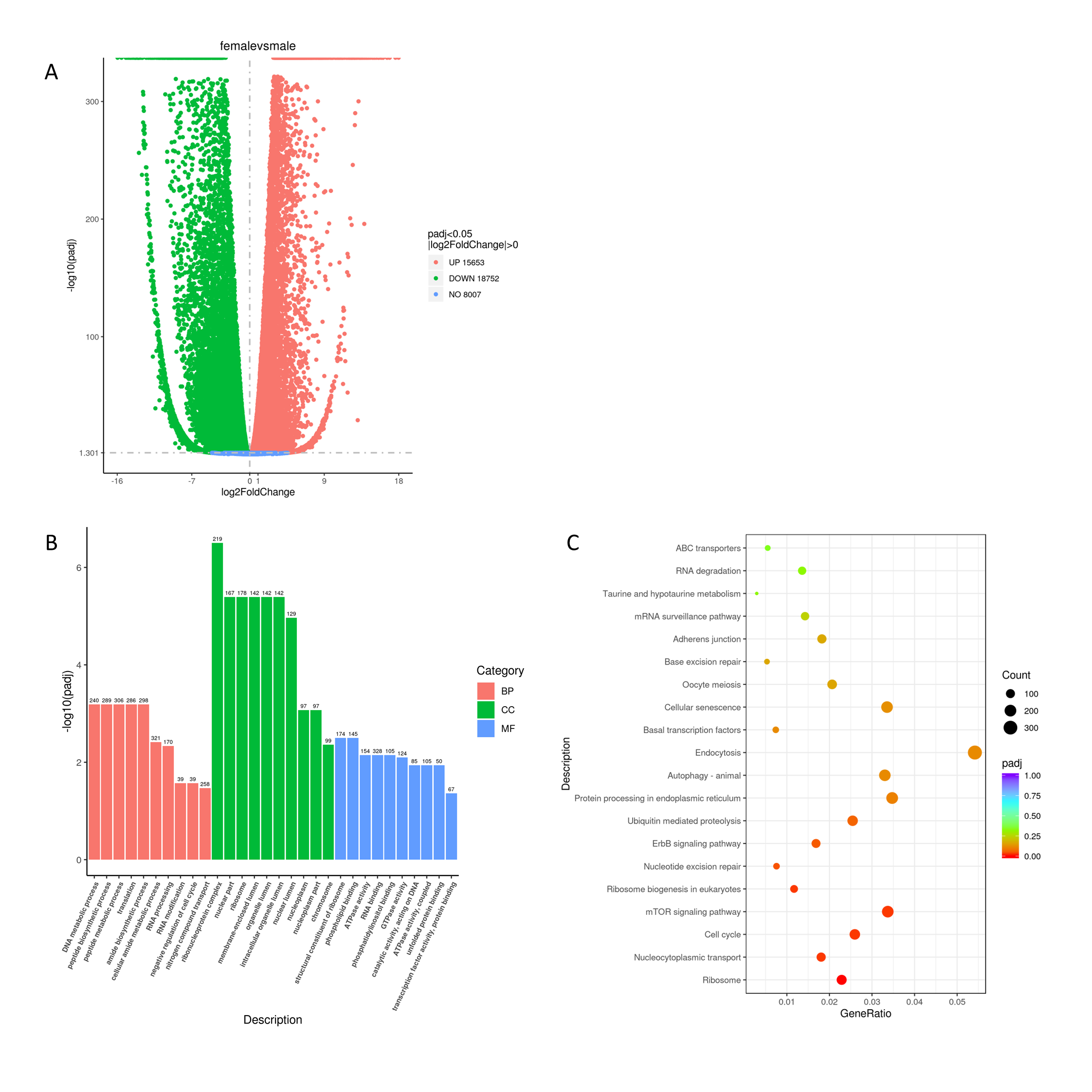

Supplement: Supplementary file 1 [file cells-12-02631-s001.zip › Figure S3. Analysis of DE mRNAs between ovaries and testes.tif]

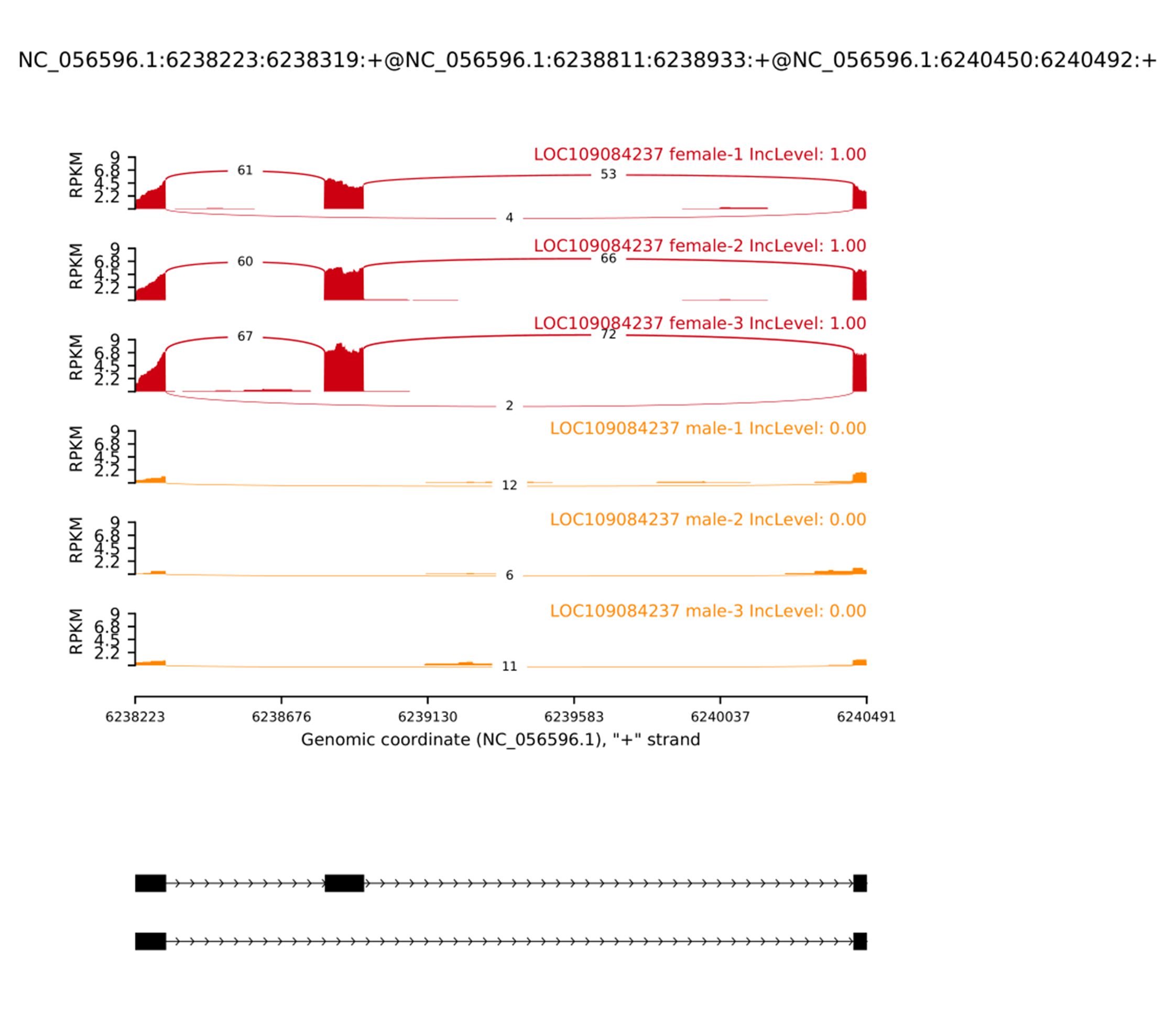

Supplement: Supplementary file 1 [file cells-12-02631-s001.zip › Figure S4. AS pattern of akap13 gene with sequence coverage depth.tif]
